# Measuring foot and ankle kinematics using a bi-plane fluoroscopic system

**DOI:** 10.1186/1757-1146-7-S1-A46

**Published:** 2014-04-08

**Authors:** Seungbum Koo, Kyoung M Lee

**Affiliations:** 1 School of Mechanical Engineering, Chung-Ang University, Seoul, South Korea; 2 Department of Orthopedic Surgery, Seoul National University Bundang Hospital, Seongnam, South Korea

## Background

Accurate measurements of skeletal kinematics of the foot would increase our understanding on the interaction between foot and footwear. Previously foot kinematics was measured using reflective markers but the method had inherent limitation of skin marker-based methods. Recently fluoroscopic imaging-based methods has been developed and widely used to measure knee kinematics [1,2]. We have made a bi-plane fluoroscopic imaging system and a walkway where continuous foot X-ray images could be taken during walking. The objective of the study was to understand the possibility of bi-plane fluoroscopic system for measuring foot and ankle kinematics.

**Figure 1:**
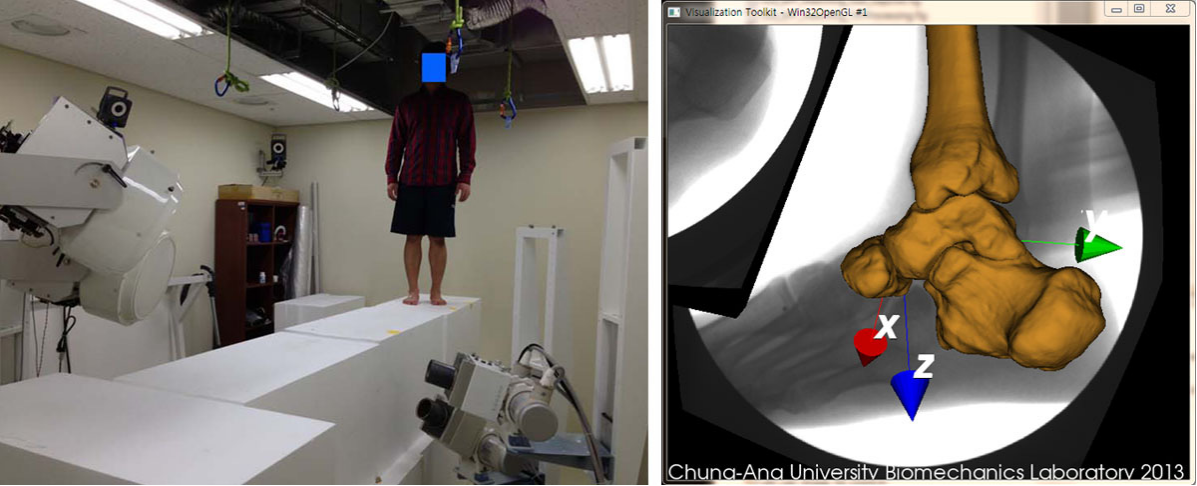
Bi-plane fluoroscopic imaging system and walking (left) and the calculation results for the positions and orientations of talus, calcaneus, navicular and tibia (right)

## Methods

The walkway was made of high density polystyrene foam, whose size was 1.2 m, 0.6 m and 4.0 m for height, width and length, respectively. The target foot position was marked on the walkway and the directions of the two X-ray imaging systems were carefully determined to capture the silhouettes of talus, calcaneus, navicular and tibia during the stance phase of walking. The study was approved by the IRB at Chung-Ang University. An informed consent was obtained from each volunteer prior to testing. Subjects walked on the walkway at their self-selected normal speed and the bi-plane fluoroscopic images were taken for 2 seconds. Images for calculating geometric calibration of the imaging system were taken. The subjects underwent computed tomographic (CT) imaging and three-dimensional bone models were obtained. A semi-automatic and manual registration methods were used to determine the positions and orientations of the four target bones for each frame of the bi-plane images during the stance phase of walking. The quality of silhouettes of the bones varied throughout the stance phase.

## Results

Ten subjects (age 21.5±1.9, all males, BMI 21.7±1.9) volunteered for the study. The registration results showed some degrees of vibration of the bones, motion noise. The general trend of foot and ankle kinematics could be observed.

## Conclusions

Foot and ankle skeletons could be imaged during the stance phase of walking using a bi-plane fluoroscopic system set-up along a polystyrene foam walkway. The positions and orientations of the foot and ankle bones could be calculated from the bi-plane images but the results showed some degrees of noise in their motions.
